# Outcomes of Preimplantation Genetic Testing for Single Gene Defects in a Privately Funded Period and Publicly Funded Period: A North-American Single Center Experience

**Published:** 2020

**Authors:** Talya Shaulov, Li Zhang, Jin-Tae Chung, Weon-Young Son, William Buckett, Asangla Ao

**Affiliations:** 1- MUHC Reproductive Centre, Department of Obstetrics and Gynecology, Montreal, Quebec, Canada; 2- Department of Human Genetics, McGill University, Montreal, Quebec, Canada; 3- McGill University Health Centre Research Institute, Montreal, Quebec, Canada

**Keywords:** In vitro fertilization, Preimplantation genetic testing, Public funding, Single embryo transfer, Single gene defect

## Abstract

**Background:**

The purpose of this study was to assess whether the outcomes from IVF-preimplantation genetic testing (IVF-PGT) cycles for single gene defects (SGD) (PGT-M) differ between a privately funded period (PRP) and publicly funded period (PUP).

**Methods:**

A retrospective cohort study was conducted in a North-American single tertiary center. The PRP (March 1998 to July 2010) comprised 56 PGT-M cycles from 58 IVF cycles in 38 couples, and the PUP (August 2010 to May 2015) comprised 59 PGT-M cycles from 87 IVF cycles in 38 couples. One PGT-M cycle is defined as one biopsy procedure from one or serial IVF cycles. A p-value of 0.05 was considered statistically significant.

**Results:**

The clinical pregnancy rates (CPR) per PGT-M cycle were 30.4% and 52.5% in each period, respectively (p=0.021). The live birth rates (LBR) per PGT-M cycle were 21.5% versus 40.9% in each period, respectively (p=0.037). A sub-analysis within the PUP comparing 39 PGT-M cycles from 39 IVF cycles with 20 PGT-M cycles from 49 IVF cycles yielded CPRs per PGT-M cycle of 64.1% and 30.0% and LBRs per PGT-M cycle of 53.8% and 15.0%, in each group, respectively (p< 0.05 for both).

**Conclusion:**

The transition from private to public funding and a single embryo transfer (ET) guideline has little impact on embryological and clinical outcomes of PGT-M cycles, and results in lower rates of multiple pregnancies. However, these two systems may serve different populations.

## Introduction

Public coverage of assisted reproductive technologies (ARTs), including *in vitro* fertilization (IVF), along with a single embryo transfer (SET) legislation, was implemented on August 5, 2010 in the province of Quebec, Canada. This program allowed for a dramatic drop in multiple pregnancy rate resulting from IVF, from over 25% to under 10% (1, 2). A SET policy has also been applied to and studied in a preimplanta-tion genetic diagnosis (PGT-M) population (3). This latter study showed that survival of cryopreserved biopsied embryos as well as implantation and clinical pregnancy rates from these embryos in PGT-M cycles was similar to these same out-comes from non-biopsied embryos in conventional IVF/ICSI cycles.

The use of PGT-M by PCR was first reported in 1990 to determine the sex of embryos in cases of X-linked recessive disorders (4). Today, PGT is classically performed for single gene disorders (SGDs) or monogenic diseases (PGT-M) as well as for structural chromosomal abnormalities (PGT-SR), as an alternative to prenatal diagnosis (5), using FISH (Fluorescence in situ hybridization), and other new cytogenetic techniques, such as aCGH (Array comparative genomic hybridization), qPCR (Quantitative real-time PCR), SNP (Single nucleotide polymorphism) microarray and NGS (Next generation sequencing), which allow for CCS (Comprehensive chromosome screening) (6–10). Depending on the jurisdiction, PGT can either be covered by private or public funding, or not covered at all.

Several studies have examined the differences in ART practice and treatment outcome between public and private clinics (2, 11–13). These studies generally show similar trends in outcomes, with higher clinical pregnancy and live birth rates in a private setting, and lower multiple pregnancy rates in a public setting. A theory that has been used to explain this phenomenon is that patients pursuing treatments in public clinics may have a worse prognosis due to the longer waiting lists in these clinics and the presence of infertility-associated risk factors linked to their lower socio-economic level (12). To our knowledge, no study to date has looked specifically at the difference in outcomes for IVF-PGT cycles between these two types of practices. Studying the effect of different funding patterns in IVF allows a comparison of outcomes, and may help expose differences in populations being treated as well as in the way cycles are managed. Through concentrated study of the issue in a PGT-M population, a more direct comparison can be achieved as the indication for treatment is the same for all patients.

The aim of this study was to compare the overall embryological and clinical outcomes of IVF-PGT cycles for SGD during the privately funded period (PRP) and the subsequent publicly funded period (PUP) in one center, in a system where only one type of funding was available at a time.

## Methods

### Study design:

A retrospective cohort study was conducted using all 115 IVF-PGT cycles performed for PGT-M between March 1998 and May 2015 in a single academic fertility center. Table 1 includes details of all PGT-M treated cases during the PRP and PUP.

**Table 1.** List of monogenic diseases for which IVF-PGT-M cycles was performed between 1998 and 2015, and classified by funding period

**Type of disease**	**PRP (March 1998 – July 2010)**				**PUP (August 2010-May 2015)**			

	**No. of cycles**	**No. of patients**	**No. of pregnancy cycles**	**No. of live birth**	**No. of cycles**	**No. of patients**	**No. of pregnancy cycles**	**No. of live birth**
**Autosomal dominant diseases**	22	14	7	8	31	18	14	12
**Autosomal recessive diseases**	27	20	8	10	18	13	9	9
**X-linked recessive diseases**	3	2	1	2	3	3	2	2
**X-linked dominant diseases**	4	2	0	0	9	6	6	5
**Total**	56 ^#^	38	16	20	61 ^*^	40 ^*^	31	28

# In PRP period, one cycle with beta Thalassemia and HLA was done in one couple.

* In PUP period, two PGT cycles with CF and DM1 were done in one couple

The two comparison groups were: (a) cycles performed from March 1998 to August 5, 2010 under private funding, and (b) cycles performed from August 5, 2010 until May 2015, under public funding. During the PRP, there were 56 PGT-M cycles performed from 58 IVF cycles (Table 2) for SGD on 38 couples in 20 different conditions (22 cycles for autosomal dominant defects in 14 couples; 27 cycles for autosomal recessive defects in 20 couples; 3 cycles for X-linked recessive defects in 2 couples; 4 cycles for X-linked dominant defects in 2 couples) (Table 1).

**Table 2.** Comparison of outcomes between the privately funded period (PRP) and publicly funded period (PUP)

**Outcome variable**	**PRP (March 1998 – July 2010)**	**PUP (August 2010 – May 2015)**	p-value ^†*^
**Embryological and diagnostic aspects**			
	n or LS mean (95%CI)	n or LS mean (95%CI)	
No. PGT-M cycles (no. patients)	56 (38)	59 (39)	N/A
No. IVF cycle	58	87	N/A
No. of oocytes collected per PGT-M cycle ^1^	15.3 (12.8, 17.9)	18.8 (16.3, 21.3)	0.048
No. of matured oocytes per PGT-M cycle ^1^	13.3 (11.3, 15.3)	14.4 (12.4, 16.4)	NS
No. of fertilized embryos per PGT-M cycle ^1^	11.5 (9.9, 13.2)	10.9 (9.3, 12.5)	NS
Fertilization rate per PGT-M cycle ^1^	0.87 (0.80, 0.94)	0.76 (0.70, 0.82)	0.019
No. of embryos biopsied per PGT-M cycle ^1^	8.4 (7.4, 9.5)	7.9 (6.9, 9.0)	NS
No. of embryos successfully tested per PGT-M cycle ^1^	7.2 (6.2, 8.2)	7.5 (6.5, 8.4)	NS
Rate of embryos successfully tested ^1^	0.85 (0.77, 0.94)	0.96 (0.87, 1.05)	NS
**Clinical outcomes**			
Average female age ^1^	31.8 (30.6, 32.9)	34.7 (33.6, 35.8)	<0.001
No. of embryos transferred per ET cycle ^3^	2.05 (1.90, 2.21)	1.15 (1.02, 1.27)	<0.0001
Implantation rate ^3^	0.24 (0.16, 0.35)	0.38 (0.27, 0.53)	NS
CPR per ET cycle ^2^	31.4% (20.0%, 45.5%)	41.8% (30.9%, 53.7%)	NS
CPR per PGT-M cycle ^2^	30.4% (19.5%, 43.9%)	52.5% (39.6%, 65.2%)	0.021
No. of live births	12	24	N/A
LBR per ET cycle ^2^	21.0% (12.0%, 34.2%)	31.1% (21.8%, 42.1%)	NS
LBR per PGT-M cycle ^2^	21.5% (12.3%, 34.9%)	40.9% (28.4%, 54.6%)	0.037

PGT-M cycle: One biopsy procedure for one or serial IVF cycles. IVF cycle: Oocyte retrieval cycle; ET cycle refers to an embryo transfer cycle; CPR: Clinical pregnancy rate; CPR per PGT-M cycle: Number of PGT-M cycles culminating in ≥ 1 clinical pregnancy divided by total number of PGT-M cycles performed. No. of sacs: Total number of sacs seen on ultrasound from all embryo transfers.

† p-value of group difference for linear regression models, groups odds ratio for logistic models or rate ratio for Poisson regression models;

* Only p-values with less than 0.05 is considered as statistically significant. NS: Not statistically significant; N/A: Not applicable;

1 - Linear regression models has been applied;

2 - Logistic models has been applied;

3 - Poisson regression models has been applied

During the PUP, a total of 59 PGT-M cycles were performed from 87 IVF cycles (Table 2) for SGD in 39 couples in 23 different conditions (31 cycles for autosomal dominant defect in 18 couples; 18 cycles for autosomal recessive defects in 13 couples; 3 cycles for X-linked recessive defects in 3 couples; and 9 cycles for X-linked dominant defects in 6 couples). There was one couple with two different diseases (DM and CF), and was calculated separately as autosomal dominant and autosomal recessive diseases.

The primary measured outcome was live birth rate (LBR) per embryo transfer (ET) and per PGT-M cycle. A live birth was defined as birth of a live fetus at or after 24 weeks of gestation. Secondary outcomes included embryological outcomes of the cycle (Oocyte maturity rates; fertilization rates; rates of embryos biopsied; and rates of embryos successfully tested), as well as clinical outcomes (Implantation rates and clinical pregnancy rates). A clinical pregnancy was defined by the presence of a gestational sac during the 5–6 weeks of gestation observed on ultrasound. The primary and secondary outcomes were calculated per PGT-M and/or per ET cycle where it was appropriate.

A subgroup analysis was performed between PGT-M cycles that comprised only one IVF cycle (Group A), and those that comprised two or more serial IVF cycles (Group B), both taking place during the PUP. The decision to perform one versus serial IVF cycles was a shared decision between the treating physician, the patient and PGT laboratory staff, mostly depending on whether there were sufficient embryos for biopsy.

### Details of IVF cycle protocol:

In the IVF cycles included in this study, women were treated with gonadotropins (Recombinant FSH, LH or hMG) and GnRH agonists or antagonists in short or long protocols. Cycle monitoring was performed by ultrasound for follicular assessment as well as by estradiol measurements. A single dose of 10, 000 *IU* hCG was administered when 1 or more follicles measured at least 17 *mm* by ultrasound. Triggering with GnRH agonist was done only in GnRH antagonist protocols if the patient was judged to be at risk of Ovarian Hyper-Stimulation Syndrome (OHSS). Oocyte retrieval was performed 36 *hr* after the hCG or GnRH agonist trigger. ICSI was performed in all cycles.

Prior to implementation of public funding in August 2010, embryos were generally biopsied and transferred in a fresh cycle, unless embryo freezing was clinically indicated for situations like OHSS, or unless a technical problem was encountered. After the implementation of public funding, serial freezing of embryos over several cycles of IVF for PGT-M was used more frequently due to lack of oocytes retrieval per stimulation cycle. The technique for embryo cryopreservation switched from slow freezing to vitrification in 2005 in this laboratory. An IVF cycle is defined as a stimulation cycle that culminated in an oocyte retrieval; and a PGT-M cycle is defined as one biopsy procedure performed for one or serial IVF cycles.

### Biopsy details:

All embryos used in this study were biopsied on day 3. Embryos frozen on day 2 were cultured for an additional day after thawing before blastomere biopsy was performed as described previously (14). Embryos frozen on day 3 were thawed in the morning and biopsied on the same day after culturing for a few hours for equilibration. Briefly, each embryo was put in a droplet of Ca^2+^ and Mg^2+^ -free medium (Cook Canada Inc.), and a hole (15–20 *μm* in diameter) was drilled in the zona pellucida using 1.48 *μm* infrared diode laser in computer controlled non-contact mode (Hamilton Thorn Inc., MA, USA). One blastomere from each embryo was aspirated gently. After removal, each blastomere was washed three times with PBSA (Phosphate-buffered saline with 4 *mg/ml* bovine serum albumin, Sigma Inc., Canada) before being used in PCR. Each biopsied embryo was washed three times with embryo culture medium and placed in a clean labeled medium under oil. Embryos were incubated in separate dishes. For PCR-based genetic analysis, washed blastomeres were transferred into a 0.2 *ml* PCR thin wall tube containing 5 *μl* alkaline lysis buffer (200 *mM* potassium hydroxide and 50 *mM* DTT). A sample of PBSA from the last wash was taken as a blank to test for contamination. Unique nested or semi-nested multiplex PCR protocols involving fluorescently labeled primers specific for mutated alleles and STR markers were developed specifically for each couple. The PCR products were analyzed on ALFexpress Automated DNA Sequencer (Amersham Pharmacia Biotech, USA) or ABI 3130 Genetic Sequencer (Applied Biosystems Inc, USA). The GeneMapper software was used to analyze the data (Applied Biosystems Inc, USA). The embryos diagnosed as unaffected were transferred into the patients 4, 5, 6 days after fertilization. Surplus normal embryos were cryopreserved at this stage.

### Statistics:

The difference between private and public coverage period was accessed by the linear or generalized linear mixed models (15, 16) with two levels of clustering (IVF-ET cycle within PGT-M cycle and PGT-M cycle within woman) separately for each available woman; PGT-M cycle and IVF cycle characteristics were indicated as outcomes and coverage type as predictor. These statistical methods allow us to take into account different types of outcomes with potential correlation across multi-level structure of the data and obtain more precise estimate than simple t-test or chi-squared test. The linear, logistic or poisson regressions with random effects has been applied for continuous, binary or count outcomes, respectively. To compare single IVF-ET and multiple IVF-ET cycles during the public coverage period, the similar approach described above has been used. All tests were conducted with a two-sided 0.05 level of significance, least squared mean (LS mean) estimates, odds ratios, and 95% confidence intervals of estimates were computed from the models. SAS version 9.4 software (SAS Institute, NC) was used for all calculations.

The Research Ethics Board of Royal Victoria Hospital at the McGill University Health Center approved this project.

## Results

The analysis was performed on a total of 144 ET cycles which corresponds to 115 PGT-M cycles or 71 unique women. The breakdown of these cycles was detailed in the materials and methods section and is presented in table 1.

### Global outcomes:

Table 2 displays the embryological and diagnostic outcomes of the PGT-M cycles. The average age of patients in the private period was lower than that in the public period (31.8 *vs*. 34.7 years old, respectively; p<0.001). The number of mature eggs per PGT-M cycle and fertilization rate was similar in the PRP and PUP groups. There was an average of 8.4 embryos biopsied per PGT-M cycle in the PRP group, and 7.9 in the PUP group, and an average of 7 embryos successfully tested per PGT-M cycle in both groups. There were113 embryos transferred in the PRP group, and 67 were transferred in the PUP group during the first transfer. There were a total of 37 embryos transferred in 30 subsequent transfers.

There were more IVF cycles performed per PGT-M cycles in the PUP group than in the PRP group, with a total of 87 IVF cycles performed for 59 PGT-M cycles and 58 IVF cycles performed for 56 PGT-M cycles in each of the groups, respectively. The average number of embryos transferred per ET cycle was significantly higher in the PRP than PUP group (2.05 versus 1.15, p<0.0001). The 38% implantation rate in the PUP group was not significantly higher than the 24% implantation rate observed in the PRP group. The clinical pregnancy rates (CPR) per PGT-M cycle and per ET were 30.4% and 31.4% respectively, in the PRP group; and 52.5% and 41.8% respectively, in the PUP group. The CPR per PGT-M cycle was significantly different between the two groups. There were 20 live births during the PRP, including one set of triplets and 6 sets of twins (Multiple birth rate per delivery, 58.3%) and 24 live births during the PUP, including one set of twins (Multiple birth rate per delivery, 4.2%). The live birth rate (LBR) per PGT-M cycle and per ET cycle was 21.5% and 21.0%, in PRP group, respectively and 40.9% and 31.1% in the PUP group, respectively. The difference was significant for LBR per PGT-M cycle.

### Subgroup comparisons:

During the PRP, all but two PGT-M cycles involved only one IVF cycle. Two serial IVF cycles were done for each of these two mentioned PGT-M cycles. During the PUP, 39 PGT-M cycles in 28 couples involved only one IVF cycle, whereas 20 cycles in 17 couples involved two or more serial IVF cycles. Five couples had overlap in both of these groups, as some couples had more than one PGT-M cycle performed for them.

Table 3 displays the comparison in outcomes between PGT-M cycles that comprised only one IVF cycle (Group A), and those that comprised two or more serial IVF cycles (Group B), both taking place during the PUP. There were 39 IVF cycles that took place in group A, and 49 IVF cycles that took place in group B. Group B showed a higher average female age than group A (34.6 *vs*. 32.9 years old, p=0.0036). The average number of total oocytes and mature oocytes collected per PGT-M cycle was significantly higher in group B than group A (26.3 *vs.* 15.5 and 18.9 *vs*. 12.4, respectively). However, the number of embryos biopsied and number of embryos successfully tested was similar. The total number of embryos transferred in group A was two times more than that in group B (65 *vs.* 35, respectively); this was proportional to the number of PGT-M cycles in group A as being two times more than group B. The number of embryos transferred at a time was not significantly different between groups: 1.10 in group A, and 1.24 in group B (p=0.1371). In group A, there were 21 live births (Including 1 set of twins) and in group B there were 3 singletons born. The LBR per ET was 40.7% and 10.7% in group A and group B, respectively, which was significantly different. The LBR per PGT-M cycle was 53.8% in group A and 15.0% in group B, which was also significantly different.

**Table 3.** Comparison between PGT-M cycles comprising one IVF cycle (group A) and PGT-M cycles comprising ≥ 2 serial IVF cycle (group B), both during the PUP (August 2010 – May 2015)

**Outcome variable**	**Group A**	**Group B**	p-value ^†*^
**Embryological and diagnostic aspects**			
	n or LS mean (95%CI)	n or LS mean (95%CI)	
No. PGT-M cycles (no. patients)	39 (28)	20 (17)	N/A
No. of IVF cycles	39	49	N/A
No. of oocytes collected per IVF cycle ^1^	15.5 (12.4, 18.6)	26.3 (22.0, 30.5)	<0.001
No. of mature oocytes per IVF cycle ^1^	12.4 (10.0, 14.8)	18.9 (15.5, 22.2)	0.003
No. of fertilized embryos per IVF cycle ^1^	10.1 (8.3, 11.8)	12.5 (9.9, 15.0)	NS
Fertilization rate per PGT-M cycle ^1^	0.81 (0.73, 0.91)	0.68 (0.60, 0.78)	0.038
No. of embryos biopsied per PGT-M cycle ^1^	7.6 (6.6, 8.7)	8.3 (6.8, 9.8)	NS
No. of embryos successfully tested per PGT-M cycle ^1^	7.3 (6.2, 8.3)	7.8 (6.3, 9.3)	NS
Rate of embryos successfully tested ^1^	0.97 (0.86 1.10)	0.93 (0.78, 1.19)	NS
**Clinical outcomes**			
Average female age ^1^	32.9 (31.5, 34.3)	34.6 (33.1, 36.2)	0.004
No. of embryos transferred per ET cycle ^3^	1.10 (1.00, 1.21)	1.24 (1.09, 1.38)	NS
Implantation rate ^3^	0.46 (0.31, 0.68)	0.20 (0.09, 0.44)	NS
CPR per ET cycle ^2^	49.7% (35.9%, 63.5%)	25.3% (11.8%, 46.2%)	0.053
CPR per PGT-M cycle ^2^	64.1% (47%, 78.2%)	30.0% (13.4%, 54.3%)	0.026
No. of live births	21	3	N/A
LBR per ET cycle ^2^	40.7% (28.5%, 54.1%)	10.7% (3.3%, 29.6%)	0.014
LBR per PGT-M cycle ^2^	53.8% (37.3%, 69.6%)	15.0% (4.5%, 39.6%)	0.014

* Only p-values with less than 0.05 is considered as statistically significant. NS: Statistically not significant. N/A: Not applicable

† P-value of group difference for linear regression models, groups odds ratio for logistic models or rate ratio for Poisson regression models

1 - Linear regression models has been applied;

2 - Logistic models has been applied;

3 - Poisson regression models has been applied

## Discussion

This study compares embryological and clinical outcomes in IVF-PGT-M cycles for SGD between a privately funded period and a subsequent publicly funded period, and to our knowledge, is the first study to do so. The common indications for PGT-M in our database, such as cystic fibrosis, fragile X syndrome, Huntington disease and myotonic dystrophy type I, are consistent with common indications cited in the literature (17, 18).

In comparing results from our study to the data presented by the European Society of Human Reproduction and Embryology (ESHRE) (PGT-M Consortium in 2015), among a large cohort of 9,267 oocyte retrieval (OR) cycles for monogenic diseases performed up to 2010 and in which most biopsies were performed on day 3 (18), similar embryological outcomes across all groups can be observed. Clinical pregnancy rate per ET was 36%, which falls between the values of our PRP (31.4%) and PUP (41.8%) groups. LBR per ET was 31%, which resembles the 31.1% LBR in our PUP. There was no distinction between public and private clinics, therefore it is difficult to compare the ESHRE data head to head with our data; however, with its robustness in numbers, it can be used to validate ours.

The lower number of embryos transferred at a time in the publicly funded group is explained by the legislated SET policy that was implemented with the public program. This pattern has also been observed in other studies examining different practices between publicly and privately funded systems (11). As expected, the SET policy also brought with it lower rates of multiple births, a pattern which has been widely observed in the literature (1, 19–26).

There were 58 IVF cycles performed for 56 PGT-M cycles during the PRP, and 87 IVF performed for 59 PGT-M cycles during the PUP. This indicates that more serial stimulation and freezing cycles were performed during the PUP to attain enough and a similar number of embryos for one PGT-M cycle. This discrepancy could reflect an underlying difference in the two patient populations that was introduced by public funding, explained by several theories. Firstly, when patients were funding their own treatments, clinicians prescribing the treatment may have been more aggressive with stimulation; or it may be that only cycles resulting in adequate follicular response proceeded to oocyte retrieval. Another possibility is that the populations in the two groups are different in their response to treatment, suggesting lower ovarian response in the PUP group. It is to note that when public funding in Quebec was implemented, demand for IVF for PGT-M as well as for all other indications rose substantially (13). This created a wait list for PGT-M (Approximately two years), which may have influenced patients’ response to stimulation as a function of their age.

The implantation rate in the PUP group was 14% higher than the one in the PRP group, despite having a lower average number of embryos transferred per ET cycle, although not statistically significant. One hypothesis to explain this difference could be that in the PUP group, there were more frozen ET cycles (in PUP, >30% of embryos transferred were frozen, and in PRP, 10% were frozen), thereby creating a selection of better quality embryos for transfer. Also, only embryos of good quality are cryopreserved; however, in fresh cycles, when there are no other embryos to transfer, average quality embryos can be transferred. This pattern is in contrast to the observations by Castilla et al. (12), who studied the differences in practices and outcomes in ART between private and public clinics in Spain, not exclusively from PGT-M cycles. This latter study showed a higher implantation rate in all years studied in the private clinics. This study also showed significantly higher CPRs per cycle and per ET among the private clinics than among public clinics. This pattern is the reverse of the one in our study, which showed higher CPRs during the PUP; however, the difference was only significant for CPRs per PGT-M cycle and per patient in our study despite a SET policy. It is possible that certain differences between the private and public clinics in Spain may render better quality embryos for transfer in private clinics. For example, as mentioned earlier, patients undergoing treatment in private clinics may have a better prognosis than those in public clinics (27); or if there is a higher rate of preimplantation genetic testing for aneuploidy (PGT-A) performed in private clinics, this may create a bias in the pregnancy rates. Also of note is that in Castilla et al.’s study (12), 96% of oocyte donation cycles and 99% of PGT-M cycles were performed in private clinics, which also may have influenced the superior results observed among these clinics.

In our main outcome of LBR, the most clinically useful comparison is the LBR per PGT-M cycle between the two groups. This is because there were more stimulation cycles per PGT-M cycle performed in the PUP, and there was a shift in embryo transfer policy that reduced the number of embryos transferred per cycle significantly in the PUP. There was a LBR per PGT-M cycle of 21.5% during the PRP, and 40.9% during the PUP (p=0.037); this difference is similar to the difference in CPRs per PGT-M cycle between the two groups.

Also, the two seemingly heterogeneous populations were analyzed: those requiring only one IVF cycle per PGT-M cycle and those requiring more than one IVF cycle per PGT-M cycle (Table 3). This analysis was limited to the PUP because during the PRP, all but two PGT-M cycles were the result of only one IVF cycle; however, during the PUP, approximately in one third of the PGT-M cycles (20/59) performed, two or more IVF cycles were observed. As expected, the latter distinct group of patients requiring serial IVF cycles for one PGT-M cycle experienced worse clinical outcomes. The LBRs and CPRs per PGT-M cycle are significantly lower and the CPR per ET is almost significantly lower in the group of patients requiring serial IVF cycles. Along with the theories mentioned above that could differentiate these two populations, the worse clinical outcomes demonstrated in this latter group of patients may be the result of the older mean age of patients in this group (34.6 *vs.* 32.9 years old), or may raise the possibility of underlying infertility as well in this group.

When comparing results of PGT-M cycles in the PRP with results of patients in the PUP who had only one IVF cycle per PGT-M (Group A), and thereby removing the so-called “poor prognosis” patients (Group B) from the analysis, there were even more pronounced improvement in clinical outcomes in group A of the PUP than PRP (Data not shown). This is in contrast to published literature comparing outcomes in privately and publicly funded programs in the same time period (11, 12), and could suggest that laboratory practices and conditions improved over the years. Also, the so-called “poor-prognosis” patients were removed from the PUP, but not from the PRP, even though these patients were less likely to be present in the latter group.

Because patients undergoing IVF with PGT-M generally have fewer embryos available to transfer than their infertile counterparts undergoing conventional IVF (28), it is important to consider factors predictive of successful outcomes. LBRs have been strongly associated with oocyte yield in multiple studies regarding ART without PGT-M (29–33). Sunkara et al. (2011) (29) published a study on the largest cohort of patients, which showed that LBR increased with increasing oocyte yield up to 15 oocytes, and declined after 20 oocytes. In the PGT-M population, oocyte yield has also been associated with clinical outcomes; however, a lower yield (<7 oocytes) is still associated with reasonable outcomes and so thresholds have been discouraged in recent studies (28, 34–36). The number of embryos available for biopsy or transfer has also been found to be a predictive factor of success. A recent study on PGT-M for monogenic diseases (36) found that at least 4 embryos available for biopsy on day 3 were necessary to produce at least one unaffected embryo, and that the number of genetically unaffected embryos significantly contributed to outcome (Odds ratio=1.345). In PGT-M, however, with each type of disease having different inheritance patterns, it is difficult to comment on the number of available embryos for biopsy or transfer, as each type of disease would logically carry with it a different threshold number. In our cohort of patients in this study, the rate of unaffected embryos was 55.9% in the PRP, and 50.6% in the PUP (p=0.569). The practice of cancelling cycles with less than four embryos available for biopsy has also been discouraged as long as patients are counseled properly on lower chances of success (28).

The main limitation of our study is its retrospective design. Additionally, this study took place in two consecutive time periods, which introduces bias from the influence of changes in practice over time. The simultaneous introduction of an eSET policy with public funding as well as a shift in cryopreservation practices that ensues from this policy are considered the main modifications and improvements in this study.

In areas where these two systems exist in parallel, these two types of clinics may serve different populations, making them difficult to compare. For instance, private clinics may have better prognosis as mentioned, may have better performing laboratories or may perform certain techniques more often or practice the ones that public clinics do not, thereby offering patients better prognosis. In our study, only one type of funding was available in the province in each studied period eliminating partly but not completely the effect of heterogeneous populations between clinics. In other words, wealthier patients would likely be found in both groups. However, a public system may introduce longer waiting lists and patients of lower socioeconomic level.

## Conclusion

To our knowledge, this is the first study examining the differences in outcomes specifically in IVF-PGT-M cycles between privately and publicly funded fertility clinics. It has been shown that switching from a private to public setting and a single embryo transfer policy may affect the number of stimulation cycles necessary to obtain sufficient oocytes for one PGT-M cycle; however, clinical results (Implantation rates and LBRs) remain unaffected and are possibly even better, especially when correcting those PGT-M cycles requiring more than one stimulation cycle. Of importance as well is the reduced rate of multiple pregnancies that is introduced by a SET technique. Larger prospective studies are necessary to corroborate these results, ideally comparing similar patient populations.
